# ^1^H NMR Reveals the Mechanism of Potassium Lactate on Proteolysis and Taste Metabolites of Rugao Ham

**DOI:** 10.3390/foods12071453

**Published:** 2023-03-29

**Authors:** Xin Cai, Renyong Liao, Daodong Pan, Qiang Xia, Ying Wang, Fang Geng, Changyu Zhou, Jinxuan Cao

**Affiliations:** 1 Key Laboratory of Animal Protein Food Processing Technology of Zhejiang Province, College of Food Science and Pharmaceutical Sciences, Ningbo University, Ningbo 315211, China; 2 School of Food and Health, Beijing Technology and Business University (BTBU), Beijing 100048, China; 3 Meat Processing Key Laboratory of Sichuan Province, School of Food and Biological Engineering, Chengdu University, No. 2025 Chengluo Avenue, Chengdu 610106, China

**Keywords:** Rugao ham, potassium lactate, proteolysis, taste metabolites, metabolic pathways

## Abstract

To deepen the understanding of the effect of potassium lactate on the taste of Rugao ham, proteolysis index, enzyme activities and protein degradation of Rugao ham salted with potassium lactate (0%, 0.5%, 1%, 2%) were investigated. Metabolites of Rugao ham were identified by ^1^H nuclear magnetic resonance (NMR) spectroscopy and the metabolic pathways of the key metabolites were enriched by the Kyoto Encyclopedia of Genes and Genomes (KEGG); the relationship between taste and metabolites was assessed by partial least square discriminant analysis (PLS-DA). The hams with 2% potassium lactate showed lower cathepsin B and L activities, and higher aminopeptidase activities than that of the control group. The contents of free amino acids and organic acids significantly increased from the control to the treatment of 2% potassium lactate. PLS-DA further demonstrated that aspartate, glutamate, alanine, serine, threonine, acetate, lactate, succinate, carnosine, β-glucose and glycerol were the key metabolites to improve the taste of Rugao ham in the treatment of 2% potassium lactate. Metabolic pathways analysis further demonstrated that amino acids metabolism was the main pathway for the taste development of Rugao ham.

## 1. Introduction

Chinese Rugao ham is one of the most celebrated traditional dry-cured meat products. It is well received by consumers because of its special flavor [1]. The salting is the key process during the production of dry-cured ham, and it plays an important role on texture, flavor, microbial stability and sensory properties of the final products [2]. For the sake of ensuring the quality of the final product, Rugao ham is usually salted with a large amount of salt (10–12% NaCl), but it also may lead to high levels of salt content in the products, which will increase the intake of salt and increase the risk of hypertension and cardiovascular diseases [3]. It is necessary to reduce the addition of salt for producing healthy Rugao ham products. However, directly reducing the use of salt in the salting process of Rugao ham may cause high activities of cathepsin, leading to excess proteolysis, which could further result in the defective texture and unpleasant flavor of ham products [4]. Consequently, it is necessary to explore effective ways for reducing salt content and improving the quality of Rugao ham.

Potassium lactate is a food additive and has been used in meat products [3,5,6]; it can not only inhibit spoilage bacteria and pathogenic bacteria, but also maintain color stability, and increase cook yields and flavor [7,8]. Astruc et al. [9] found that the using of potassium lactate in beef sausage increased the salty taste. Quilo et al. [10] found that potassium lactate could improve sensory and cooking characteristics in ground beef patties. Although potassium lactate could improve the quality of meat products, there is no research to consider the effect of potassium lactate on the taste of Rugao ham.

Taste plays an important role in determining ham quality, which depends on a large number of biochemical reactions occurring during the production of dry-cured ham [11]. Protein degradation was one of the most important biochemical reactions and it produces a variety of low molecular compounds, such as small peptides and free amino acids, and these compounds were associated with the taste development of ham products [12]. Costa-Corredor et al. [2] confirmed that potassium lactate decreased proteolysis and softness of restructured ham. Fulladosa et al. [13] revealed that the treatment of potassium lactate had no negative impact on taste of restructured dry-cured hams through sensory evaluation. Most studies only evaluated the effect of potassium lactate on taste at the sensory level [3,5,6], while the variation of taste metabolites has not been investigated in detail.

In recent years, metabolites have been determined through various methods, such as liquid chromatograph mass spectrometer (LC-MS) [14], high performance liquid chromatography (HPLC) [15] and capillary electrophoresis/time of flight-mass spectrometry (CE-TOF-MS) [16]. Nuclear magnetic resonance (NMR) technology has obvious advantages in the identification and quantification of low molecular weight metabolites compared to the above identification technologies [17]. Zhou et al. [18] found that the metabolites contributing to the taste of Jinhua ham produced by a modern-processed procedure were mainly free amino acids, peptides and organic acids based on NMR technology. These indicated that NMR was an effective technology to discriminate and determine different taste compounds of ham products.

Therefore, this research was to evaluate the change of proteolysis and taste metabolites of Rugao ham salted with different levels of potassium lactate through the proteolysis index, sarcoplasmic and myofibrillar protein degradation, cathepsin and aminopeptidase activities, and to investigate the metabolites composition by NMR technology. Moreover, the relationship between metabolites and the taste of Rugao ham was further investigated through partial least square discriminant analysis (PLS-DA), which aimed at giving a helpful understanding for the effect of potassium lactate on the quality development of Rugao ham in terms of protein degradation and taste compounds, and providing valuable information to investigate the strategy in the salt reduction of Rugao ham.

## 2. Materials and Methods

### 2.1. Processing of Rugao Ham and Sample Preparation

The Rugao hams were produced by Changshou Ham Limited Company of Jiangsu Province in China. A total of 200 fresh thighs of about 8 kg from Jiangquhai native pigs were randomly divided into four groups. The manufacture procedure of Rugao ham was performed according to methods of Wei et al. [1]. The control group was salted with 6% NaCl and 0.015% NaNO_2_, without potassium lactate; three experimental groups were salted with 6% NaCl and 0.015% NaNO_2_, and different levels (0.5%, 1% and 2%) of potassium lactate, respectively. All groups were dry-cured for 40 days at 0–8 °C and 60–80% relative humidity. After salting, the hams were washed and sun-dried for 15 days (under outdoor natural conditions), then the hams were moved to the dry-ripening room and ripened for seven months under natural conditions (10–30 °C and 70–80% relative humidity). Then, the hams were post-ripened at room temperature (20–30 °C and 70–80% relative humidity) for two months. After all processes, six hams were randomly taken in each group, the *biceps femoris* of each ham was excised and sampled. The samples were vacuum packed and stored at −40 °C until analysis.

### 2.2. Proteolysis Index (PI) Analysis

The proteolysis index was assayed according to Zhou et al. [4]; ham samples (0.7 g) with 7 mL cold distilled water were homogenized at 12,000 rpm for 30 s using the XHF-D high-speed dispensator (Xinzhi Biotechnology Co., Ltd., Ningbo, China). Then, 150 μL of extract was added, 600 μL of 12.5% trichloroacetic acid to shake at 4 °C for 15 min, and then centrifuged at 2000× *g* for 10 min. The pH of supernatant (300 μL) was adjusted to about 9.0 using 2 M NaOH. Then, the solution was added to 180 μL of fluorescamine (0.6 mg/mL) dissolved in acetone, protected from light and incubated at 25 °C for 1 h. At excitation and emission wavelengths of 375 nm and 475 nm, respectively, the fluorescence of the mixture was detected. Non-specific fluorescence of the untreated samples was subtracted. Under the same conditions as above, the glycine (5–50 mM glycine) calibration curve was prepared to calculate the N-terminal α-amino content in the ham extract. At the same time, the total protein content in the ham extract was detected using a double shrinkage kit (Solarbio, Beijing, China) before trichloroacetic acid treatment. PI (%) was defined as a percentage of N-terminal α-amino content to total protein content in the ham sample, and three replicates were performed during the analysis.

### 2.3. Cathepsin Activities Assays

Cathepsin extracts were prepared according to the method of Zhao et al. [19] with slight changes. Samples (1 g) were added to 10 mL of 50 mM sodium citrate buffer (pH 5.0) containing 0.2% Triton X-100 and 1 mM EDTA, which were homogenized at 12,000 rpm for 30 s. The extracts were centrifuged at 10,000× *g* for 20 min at 4 °C. The supernatants were filtered using gauze for cathepsin activities determination. The protein content of supernatants was measured through a biuret protein assay regent kit (Sigma-Aldrich, St. Louis, MO, USA).

Cathepsin activities were assayed in 50 mM phosphate buffer (pH 6), including 4 mM EDTA, 2 mM DTT, 0.1% Brij 35 and 100 μM substrate. Arg-Arg-AMC and Phe-Arg-AMC (Sigma Chemical Co., St. Louis, MO, USA) were substrates for activities determination of cathepsin B and B + L, respectively. The reaction solution included 250 μL of the corresponding substrate solution and 50 μL of the cathepsin extracts, and then it was incubated for 20 min at 37 °C and terminated by adding 600 μL of ethanol. At the excitation wavelength of 380 nm and the emission wavelength of 440 nm, the generated fluorescence was determined. In the meantime, calibration curves were drawn with a 7-amido-4-methyl coumarin (AMC) replacing substrate for detecting fluorescence under the same condition. Cathepsin L activities were calculated by cathepsin B + L activities subtracting cathepsin B activities. Cathepsin activities were defined as the amount of enzyme required to produce 1 nmol AMC per minute per gram of proteins at 37 °C. Six replicates were performed for each enzyme assay.

### 2.4. Aminopeptidase Activities Assays

Aminopeptidase extracts were prepared according to Zhao et al. [20]. Samples (1 g) with 10 mL of 50 mM phosphate buffer (pH 7.5) contained 5 mM EGTA were homogenized at 12,000 rpm for 30 s. Then, these homogenates were centrifuged at 10,000× *g* for 20 min at 4 °C. The supernatants were filtered using gauze for protein content determination and activities determination.

Aminopeptidase activities were analyzed, based on the descriptions of Flores et al. [21] and Zhao et al. [20]. Alanyl aminopeptidase (AAP) activities were assayed in 100 mM phosphate buffer (pH 7) containing 5 mM CaCl_2_, 1 mM DTT, 0.1% Brij 35 and 100 μM Ala-AMC as substrate. Arginyl aminopeptidase (RAP) activities were assayed in 50 mM phosphate buffer (pH 6.5) containing 150 mM NaCl, 1 mM DTT, 0.1% Brij 35 and 100 μM Arg-AMC as substrate. Leucyl aminopeptidase (LAP) activities were assayed in 50 mM phosphate buffer (pH 6.5) containing 5 mM MgCl_2_, 1 mM DTT, 0.1% Brij 35 and 250 μM Leu-AMC as substrate. The reaction solution was 250 μL of the corresponding substrate solution and 50 μL of the aminopeptidase extracts. For the Arginyl aminopeptidase (RAP) assay, the enzyme extracts were diluted with extraction buffer at 1:10 before adding substrate solution to avoid interference of cathepsin H. After incubation at 37 °C for 30 min, the solution was immediately added to 600 μL of ethanol to terminate reaction. At the excitation wavelength of 380 nm and the emission wavelength of 440 nm, the generated fluorescence was measured. Meanwhile, calibration curves were drawn with AMC under the same condition. Aminopeptidase activities were defined as the amount of enzyme required to produce 1 nmol AMC per minute per gram of proteins at 37 °C. Six replicates were performed for each enzyme assay.

### 2.5. Sodium Dodecyl Sulfate Polyacrylamide Gel Electrophoresis (SDS-PAGE) of Sarcoplasmic Proteins and Myofibrillar Proteins

The sarcoplasmic proteins and myofibrillar proteins were extracted, based on the methods of Zhou et al. [4]. Biceps femoris samples (5 g) were added to 20 mL of 50 mM pH 7.5 phosphate buffer containing 1 mM EDTA, which were homogenized at 12,000 rpm for 30 s. Then, these homogenates were centrifuged at 10,000× *g* for 30 min at 4 °C, and the supernatants were collected and they were sarcoplasmic proteins. These remaining precipitates were dissolved using 20 mL of the above same phosphate buffer for homogenization (12,000 rpm, 30 s) and centrifugation (10,000× *g*, 30 min). These processes were performed three times, and the final precipitates were myofibrillar proteins. Myofibrillar protein extracts were dissolved using 0.6 M NaCl solution. The contents of extracted sarcoplasmic proteins and myofibrillar proteins were determined through a double shrinkage kit. Sodium dodecyl sulphate-polyacrylamide gel electrophoresis (SDS-PAGE) was carried out using 12% polyacrylamide separation gel and 5% polyacrylamide stacking gel to analyze the degradation of sarcoplasmic proteins and myofibrillar proteins. The gel was stained and decolorized clearly, then the protein molecular weight of each band was identified according to the protein marker. The optical density of protein bands was measured with Quantity One Imaging software (v4.6.3, Bio-Rad, Hercules, CA, USA).

### 2.6. Metabolites Extraction

Metabolites of ham samples (*n* = 6) were extracted by the method of Zhang et al. [22]. First, 1 mL of methanol/water (2:1, *v*/*v*) was used to extract ham samples (1 g) by homogenizing at 12,000 rpm for 2 min. The extracts were centrifuged for 10 min at 4 °C and 12,000× *g*. After removing the methanol by vacuum evaporation, the supernatants were lyophilized and collected. Before NMR analysis, the extract was dissolved into 1 mL of 0.15 M pH 7.4 K_2_HPO_4_/NaH_2_PO_4_ buffer, including 50% D_2_O and 0.1% sodium 3-trimethylsilypropionate [2,2,3,3-d_4_]. After centrifugation for 10 min at 4 °C and 12,000 rpm, 500 μL of supernatant was taken and transferred into an NMR tube (Φ = 5 mm) for further NMR analysis.

### 2.7. ^1^H NMR Spectroscopy

^1^H NMR spectra of the extracts were recorded on a Bruker Avance 600 MHz Spectrometer (operating at 600.13 MHz, Bremen, Germany) equipped with an ultra-low temperature detection probe at 298 K. The metabolite profile of each ham sample was collected using a standard Bruker pulse sequence NOESYGPPR1D (RD-90-t1-90-tm-90-acquisition) with a weak irradiation during recycle delay (RD, 2 s) and mixing time (tm, 100 ms). A 90° pulse length was set to 15 μs and t1 was adjusted to 2 μs. A total of 32 transients were collected into 32 k data points with a spectral width of 20 ppm. All free induction decays were subjected to an exponential window function with a line broadening factor of 0.3 Hz prior to Fourier transformation. The residual water (δ 4.5~5.0) signal in the spectra was removed. The content of metabolites was measured according to the internal standard (sodium 3-trimethylsilyl propionate). The taste-active values (TAVs) of metabolites were calculated to confirm the taste contribution, according to these descriptions [17,23].

### 2.8. Statistical Analysis

The data of PI, cathepsin B and L activities, aminopeptidase (AAP, RAP and LAP) activities and metabolites content were analyzed using one-way analysis of variance (ANOVA) at a significance level of 5% by SAS 8.1 (SAS Institute Inc., Cary, NC, USA). Duncan multiple comparison was selected to analyze the significant difference of these data. Principle component analysis (PCA) was performed to characterize the differences in metabolite components among different groups. PLS-DA was carried out to further analyze the differences in the metabolite contents among different groups using SIMCA-P version 14.1 (UMETRICS, Umeå, Sweden). Metabolic pathways associated with the key metabolites were investigated using the pathway database of the Kyoto Encyclopedia of Genes and Genomes (KEGG).

## 3. Results and Discussion

### 3.1. Changes of PI in Rugao Hams

**Figure 1:**
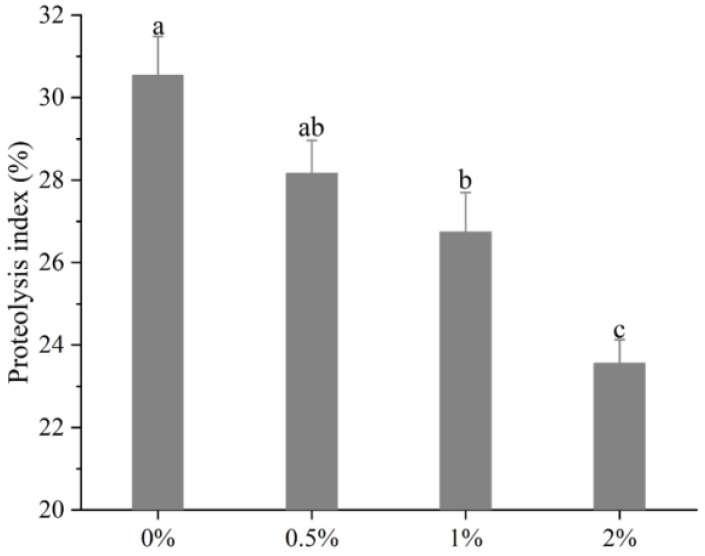
Changes of proteolysis index of *biceps femoris* muscle in Rugao hams. ^a–c^ different lowercase letters mean significant difference among four groups.

The changes of PI values of the biceps femoris muscle in Rugao ham are shown in Figure 1. It can be observed that with the increase of potassium lactate from 0% to 2%, the PI sharply decreased from 30.55% to 23.56% (*p* < 0.01), indicating that potassium lactate inhibited the proteolysis of Rugao ham. Similarly, Costa-Corredor et al. [2] also found that the application of potassium lactate reduced the PI values of restructured dry-cured hams.

The proteolysis index is an index commonly used to evaluate the degree of protein hydrolysis. During the processing of dry-cured ham, proteins were hydrolyzed to varying degrees, while moderate proteolysis played a significant part in the formation of better texture and flavor [24]. The research showed that the protein hydrolysis index of high-quality Spanish dry-cured ham was 33–36%, that of Italian ham was 22–30% [25], and PI values of Jinhua ham were between 14–20% [11]. The protein hydrolysis index of Rugao ham was usually about 22%, while the PI values measured in this study were close to those of high-quality Italian ham, indicating that moderate protein hydrolysis occurred in the processing of Rugao ham, which made the final product obtain high quality. PI values are mainly affected by raw materials and processing conditions [26]. Generally, the low pH value and salt content, and the high moisture, could increase protein degradation [27], while previous studies found that potassium lactate increased pH value and decreased moisture [13]. Therefore, potassium lactate may inhibit the hydrolysis of protein to some extent by increasing pH and decreasing moisture. In addition, abnormal growth of microorganisms also could result in the occurrence of intense protein hydrolysis [28], while potassium lactate has the antibacterial ability [8], which can prevent excessive proteolysis due to microbial activity. Thus, lower PI in the treatment of 2% potassium lactate could be attributed to the decreased activities of endogenous enzymes [24].

### 3.2. Changes of Cathepsin Activities

**Figure 2:**
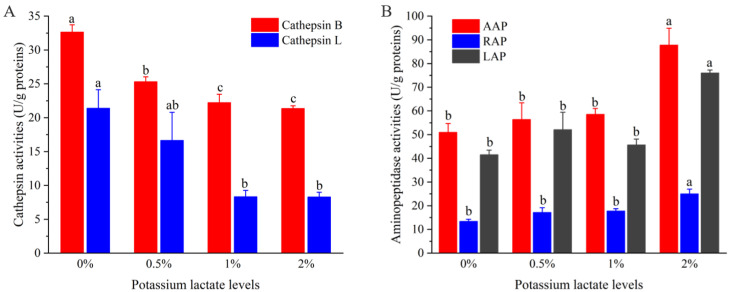
Changes of the activities of cathepsin (**A**) and aminopeptidases (**B**). ^a–c^ different lowercase letters mean significant difference among four groups.

Figure 2A shows the variations of the activities of cathepsin B and L in Rugao ham with different additions of potassium lactate. With the increase of potassium lactate from 0% to 2%, the cathepsin B activities decreased from 32.64 U/g proteins to 21.37 U/g proteins, and the cathepsin L activities also decreased from 21.40 U/g proteins to 8.29 U/g proteins. Compared with the control group, the cathepsin B and L activities in treatment of 2% potassium lactate were significantly lower (*p* < 0.05), which showed that the treatment of 2% potassium lactate significantly inhibited the cathepsin B and L activities.

Cathepsin are mainly contributed to the formation of polypeptides, and its activities are influenced by pH, salt content and temperature. In terms of pH, cathepsin maintain high activities in the range of pH 5.5–6.2. With the increase of pH values, their activities would decrease [19]. However, potassium lactate has the ability to decrease pH values [5], which may inhibit cathepsin activities to some extent. It has been reported that cathepsin B and L could play a significant part in degrading myofibrillar proteins, including actin, tropomyosin, myosin and troponin-T [29]. Furthermore, cathepsin B and L also could promote the degradation of insoluble proteins to soluble proteins, and soluble proteins to peptides [19]. Therefore, the lower cathepsin B and L activities of ham treated with 2% potassium lactate could be responsible for higher intensity of protein bands than that of control group, which will be discussed in the following.

### 3.3. Changes of Aminopeptidase Activities

Figure 2B shows the changes of aminopeptidase (AAP, LAP, RAP) activities of Rugao ham with different additions of potassium lactate. Compared with the control group and other groups, the activities of three aminopeptidases, including AAP, LAP and RAP in the treatment of 2% potassium lactate, were significantly higher, indicating that 2% potassium lactate significantly promoted aminopeptidase activities. Among the three aminopeptidases, the activities of AAP were the highest (50.96–87.82 U/g proteins), followed by LAP activities, while the RAP activities (25.03 U/g proteins) were the lowest in the treatment of 2% potassium lactate. This was in line with the results detected by Toldrá et al. [30], who found that AAP showed the highest exopeptidase activities during the production of ham, because AAP was the main aminopeptidase in skeletal muscle, accounting for about 83% of the total aminopeptidase activities [31]. Zhao et al. [20] also revealed that LAP and AAP kept high activities in the whole process of Jinhua ham.

Aminopeptidases are mainly responsible for the degradation of peptides and are conducive to the generation of free amino acids, and free amino acids could be associated with taste development of ham products and produce other volatile compounds to promote the formation of flavor [12]. Among them, AAP showed broader substrate specificity toward aromatic, aliphatic and basic aminoacyl bonds [31]. LAP processed high substrate specificity for hydrophobic amino acids [32], while RAP was more restricted to a few terminal amino acids mainly of basic nature, such as arginine and lysine [30]. Therefore, the increased aminopeptidase activities in treatment of 2% potassium lactate may result in the increase of free amino acids. In fact, the AAP, LAP and RAP activities could be promoted by elevated pH but inhibited by high salt [20]; potassium lactate can reduce salt absorption and increase pH values [5], which may be a reason for the increase of aminopeptidase activities in treatment of 2% potassium lactate.

### 3.4. SDS-PAGE Analysis

**Figure 3:**
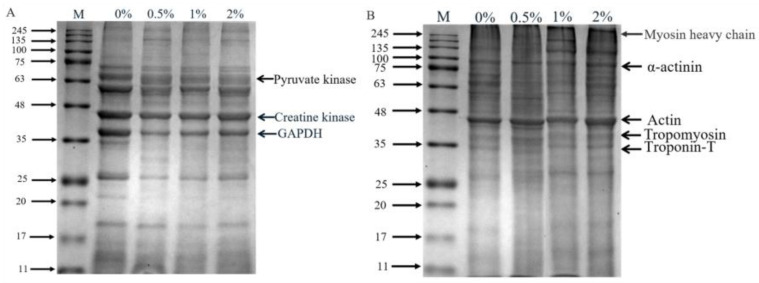
SDS-PAGE profile of sarcoplasmic (**A**) and myofibrillar proteins (**B**). Specifically, 0%, 0.5%, 1% and 2% represent potassium lactate levels. The proteins in gel were identified according to the molecular weight of standard proteins (11–245 kDa) and previous studies [4,33]. In the sarcoplasmic proteins, 63 (pyruvate kinase), 43 (creatine kinase) and 37 (GAPDH); in the myofibrillar proteins, 220 kDa (myosin heavy chain), 100 kDa (α-actinin), 43 kDa (actin), 36 kDa (tropomyosin) and 33 kDa (troponin-T).

The SDS-PAGE of extracted sarcoplasmic proteins and myofibrillar proteins is shown in Figure 3, and the optical density of protein bands is showed in Table S1 (Supplementary Materials). There were obvious differences in the protein profile of sarcoplasmic proteins (Figure 3A). Compared to the control group, the band intensity of 69 kDa and 63 kDa protein in hams with 2% potassium lactate was higher, while the intensity of 37 kDa, 26 kDa and 18 kDa protein bands in hams with 2% potassium lactate was lower. Similarly, in myofibrillar proteins (Figure 3B), compared with the control ham, ham with 2% potassium lactate showed the higher band intensity of 43 kDa and 26 kDa protein and the lower band intensity of 70 kDa and 65 kDa protein. These indicated that the degradation of myofibrillar proteins and sarcoplasmic proteins in treatment of 2% potassium lactate was lower than that of the control group.

Myofibrillar proteins and sarcoplasmic proteins are largely degraded at the ripening stage of dry-cured ham, and their degradation mainly affects the texture and taste characteristic of ham [28]. Referring to the relevant literature, 37, 43 and 63 kDa of protein bands in sarcoplasmic proteins were identified as GAPDH, creatine kinase and pyruvate kinase [4,33], respectively. Creatine kinase and pyruvate kinase were closely associated to the formation of ham flavor [34]. The studies confirmed that ham with high adhesiveness showed higher protein hydrolysis in GAPDH and creatine kinase compared to ham with low adhesiveness [35]. Similarly, in this study, the band intensity of GAPDH in the control group was higher than that in ham with 2% potassium lactate, indicating that the control ham had the more violent degradation of sarcoplasmic proteins.

Myosin, α-actinin, tropomyosin and troponin are likely to be involved in stabilizing and specifying the highly ordered structure of muscle. The myosin heavy chain (220 kDa) almost disappeared, which was demonstrated during the ripening processing of dry-cured ham. The protein bands at 33, 36, 43 and 100 kDa were identified as troponin-T, tropomyosin, actin and α-actinin [4], respectively. It was found that intense degradation of the myosin light chain, troponin-T and myosin heavy chain could lead to the excessive bitterness and adhesiveness of dry-cured ham [4]. Moreover, Skrlep et al. [35] also showed that excessive protein degradation can lead to excessive softness, mushy texture and unpleasant taste in dry-cured ham. In our study, the band intensity of α-actinin, actin, tropomyosin and troponin-T of the control group was lower than that of the group treated with 2% potassium lactate, demonstrating that the hams of the control group presented more intense degradation than that of ham treated with 2% potassium lactate. Furthermore, these results of more intense degradation of sarcoplasmic protein and myofibrillar protein in the control group could be favored by the changes of cathepsin activities.

### 3.5. ^1^H NMR Spectra of Rugao Hams

In order to evaluate the influence of potassium lactate on the composition of the methanol-water extract of ham, the extracts of ham manufactured with 0%, 1% and 2% potassium lactated were analyzed by NMR spectroscopy, and these components were further quantified. Figure S1 shows representative 600 MHz ^1^H NMR spectra (0–10 ppm), and the details of compounds are presented in Table S2. According to the NMR spectral attributions, 34 metabolites were identified, which were divided into seven categories, which included free amino acids (15), peptides (2), organic acids (6), nucleic acids and their derivatives (4), alcohol (1), sugar (1) and others (5). Zhang et al. [22] identified 33 metabolites of dry-cured hams. Compared to these studies, 26 of metabolites were the same but seven metabolites (α-glucose, ribose-5-phosphate, fumarate, histamine, histidine, hypoxanthine and glycine) were not identified in this study. The metabolites including butyrate, proline, myo-inositol, serine, arginine, uridine, formate and anserine were detected in the current study. The concentrations of free amino acids, peptides and organic acids were higher than that of nucleic acids and their derivatives, alcohol and sugar in all hams, which was similar to the results detected in boneless hams products [17].

### 3.6. Multivariate Statistical Analyses of Metabolites

**Figure 4:**
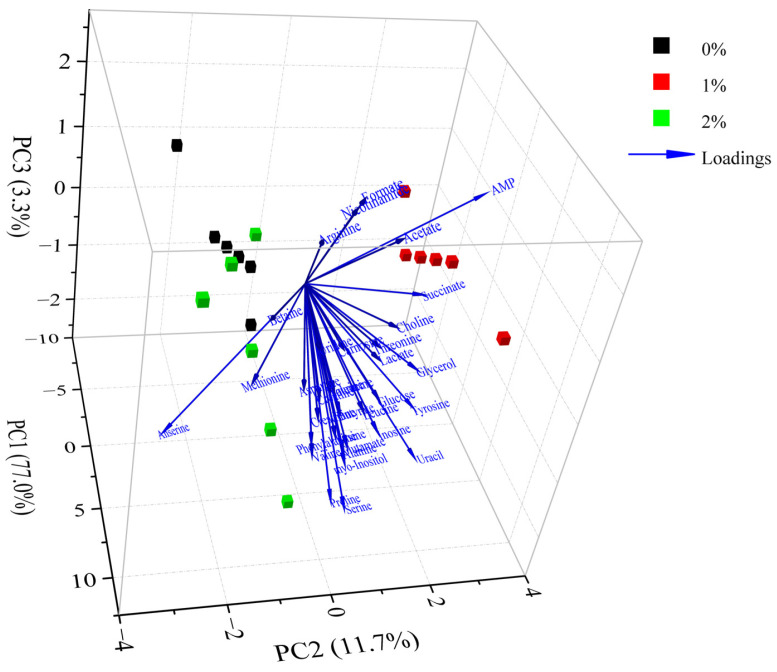
Principal component analysis of metabolites in Rugao hams treated with different levels of potassium lactate (0%, 1% and 2%).

The differences in metabolites between different groups were analyzed by PCA. According to the PCA scores plot (Figure 4), the first three principal components explained 77.0%, 11.7% and 3.3% of the total variance, respectively, which demonstrated that the sample sets with different treatments could be separated without doubt. As shown in Figure 4, the control group was noticeably discrete from these groups of 1% potassium lactate and 2% potassium lactate along with the PC1 axis, suggesting that the metabolic profile in Rugao ham was distinctly changed after treatment with potassium lactate. The group treated with 2% potassium lactate was also noticeably discrete from the group treated with 1% potassium lactate along with the PC1 and PC2 axis, demonstrating that the addition of potassium lactate also had an obvious influence on the metabolic profile of ham products. In particular, the control group was located the farthest away from treatment of 2% potassium lactate on the PC1 axis, demonstrating the most significant difference in metabolite characteristics between the two groups. All metabolites were distributed on the positive side of PC1, closest to treatment of 2% potassium lactate, which was consistent with the highest metabolite concentrations in ham treated with 2% potassium lactate among the three groups.

**Figure 5:**
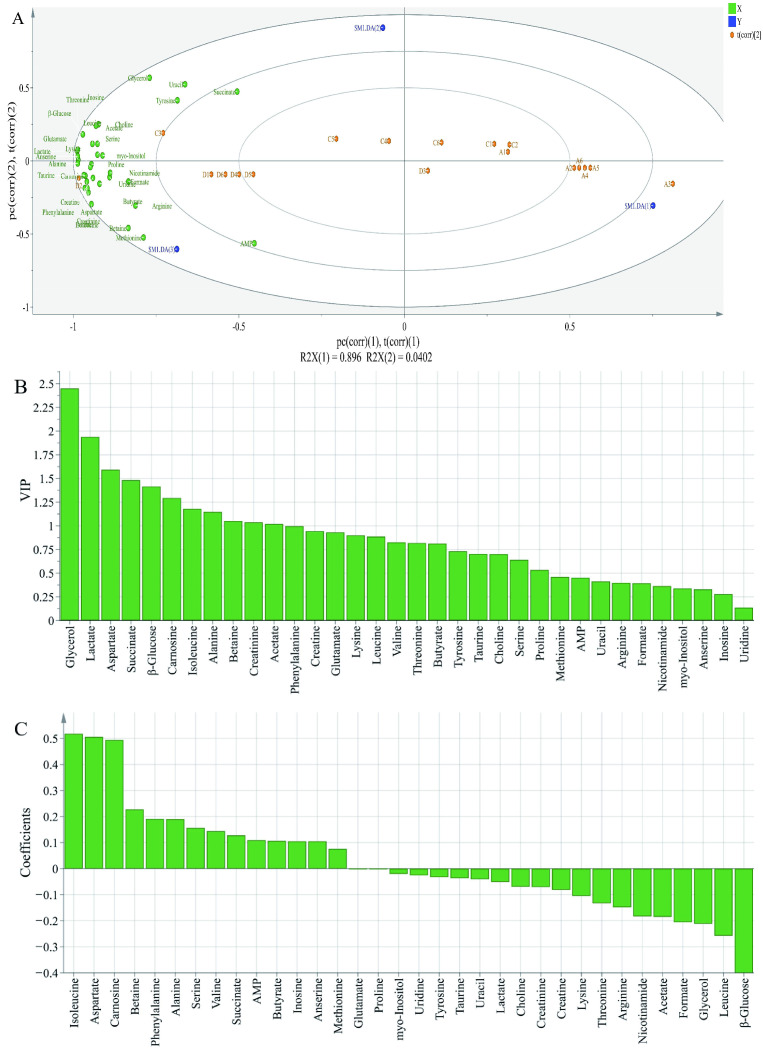
Biplot loadings (**A**), Variable Importance for the Projection (**B**) and coefficients plots (**C**) of normal and spoiled hams obtained from PLS-DA. A1-A6 represent control ham (0% potassium lactate), C1-C6 represent ham cured with 1% potassium lactate, D1-D6 represent ham cured with 2% potassium lactate.

In order to further evaluate the differences in the metabolite contents among different groups, PLS-DA analysis was performed; X-matrix was designed as the profile of metabolites and Y-matrix was set as the taste intensity derived from sensory analysis. As shown in Figure 5A, R2X was 0.979 and Q2 was 0.945, indicating that the PLS-DA model had good discrimination and predictability [36]. The PLS-DA biplot (Figure 5A) highlighted significant variations among different groups. All metabolites were close to treatment of 2% potassium lactate, but far away from the control group and treatment of 1% potassium lactate. This was because ham samples salted with 2% potassium lactate showed the highest metabolite concentrations among the three groups. Among these metabolites, tyrosine, glycerol, uracil, AMP, lactate, anserine, alanine, carnosine, aspartate, succinate, glutamate and β-glucose were closer to treatment of 2% potassium lactate, indicating that they may be the major contributors to taste of ham products. According to the Variable Importance for the Projection (VIP) shown in Figure 5B, the VIP values of glycerol, lactate, aspartate, succinate, β-glucose, carnosine, isoleucine, alanine, betaine, creatinine and acetate were higher than 1, indicating that these metabolites were considered as the key components, which helped to distinguish different groups. Therefore, these metabolites could be identified as obviously differential metabolites. Furthermore, as is shown in the coefficients diagram (Figure 5C), isoleucine, aspartate and carnosine had a positive correlation with the taste of ham with 2% potassium lactate, but β-glucose, leucine and glycerol had a negative correlation with the taste of ham with 2% potassium lactate.

### 3.7. Contribution of Metabolites to Tastes of Rugao Hams

Taste is one of the main sensory indicators to evaluate the quality of final ham products, and it is related to the composition of metabolites. In order to confirm the taste contribution of metabolites, the taste-active values (TAVs) of metabolites were calculated using the ratio of the content of each metabolite measured in Rugao ham samples to its threshold value normally reported in water or a simple matrix in these references [17,23]. According to the taste active values (TAVs) shown in Table 1, the metabolites with TAVs > 1 were considered to be the taste active metabolites in Rugao ham.

Free amino acids (FAAs) are abundant metabolites of dry-cured ham, which directly affected the taste development of ham [22]. As shown in Table 1, the content of 15 FAAs in ham with 2% potassium lactate was obviously higher than that of in the control ham (*p* < 0.05). This indicated that potassium lactate promoted the production of free amino acids, which was mainly due to the higher aminopeptidase activities in treatment of 2% potassium lactate. In all hams, alanine, glutamate, lysine, leucine, aspartate, isoleucine and valine were the most abundant FAAs and their TAVs were >1. It was found that alanine showed a sweet taste, glutamate and aspartate showed an umami taste, leucine and isoleucine showed a bitterness, lysine and valine contributed to a sweet or bitter taste depending on their concentrations [17,23]. Therefore, these FAAs were the main taste contributors to Rugao ham. Meanwhile, threonine and serine were responsible for sweetness [17], and their TAVs were less than 1 in the control group, but they were more than 1 in these ham samples treated with 2% potassium lactate, indicating that they could contribute to the taste of ham treated with 2% potassium lactate. In addition, the TAVs of methionine, arginine, tyrosine and proline were <1 in all hams, which indicated that these FAAs had little contribution to taste. Therefore, alanine, threonine, serine, aspartate and glutamate were dominant in the taste improvement of ham treated with 2% potassium lactate.

Carnosine is a histidine-derived dipeptide and anserine is a β-alanyl-1-methyl-histidine, and they were also taste active compounds in dry-cured ham. Studies have found that carnosine and anserine could increase the meaty taste and richness of meat products [23], which has a positive effect on the flavor of ham. Moreover, carnosine and anserine can inhibit the rancidity of dry-cured meat products and prolong oral taste [37]. In the present study, a higher content of carnosine and anserine was detected in treatment of 2% potassium lactate than in the control ham, which could be responsible for the taste improvement of hams with 2% potassium lactate. Furthermore, the TAVs of carnosine were >1 in treatment of 2% potassium lactate, while the TAVs of anserine were <1 in all hams, indicating that carnosine could be an important part in improving the taste of hams with 2% potassium lactate.

Organic acids also played a main part in taste attributes of dry-cured ham. A study revealed that organic acids mainly served the sour taste, but creatine presented a bitter taste [38]. In this research, the content of all organic acids significantly increased with the increase of potassium lactate from 0% to 2%. The TAVs of lactate, acetate and succinate were higher than 1, while the TAVs of creatine were lower than 1 in all hams. This showed that lactate, acetate and succinate could contribute to the taste of ham. Furthermore, succinate could reduce lipid oxidation and increase meat redness; lactate and acetate had antibacterial activity to prevent the spoilage of food [22]. These further indicated that the higher content of lactate, acetate and succinate in ham with the 2% potassium lactate than the control ham may have a positive impact on the quality of ham with 2% potassium lactate.

Nucleic acids and their derivatives may affect the taste characteristics of final products. It has been reported that AMP and inosine contributed to umami and bitterness, respectively [22]. However, the content of nucleic acids and their derivatives detected in our study were low in all hams, and the TAVs of AMP and inosine were <1, indicating that AMP and inosine were not the main taste metabolites of Rugao ham. In previous studies, it was also found that AMP and inosine were not responsible for the taste of Rugao ham products [18,22].

Studies reported that glycerol and glucose could be the contributors to the sweet taste in dry-cured ham [22]. In our research, glycerol and β-glucose were also identified, and their contents in the ham with 2% potassium lactate were higher than the control ham. In addition, the results of PLS-DA demonstrated that glycerol and β-glucose had a significant role in taste, further suggesting that the higher concentration of glycerol and β-glucose may be conducive to improve the taste of ham with 2% potassium lactate.

Combined with the results of PLS-DA and taste analysis, it could indicate that aspartate, glutamate, alanine, serine, threonine, acetate, lactate, succinate, carnosine, β-glucose and glycerol were key metabolites that could contribute to the improvement of taste in ham treated with 2% potassium lactate.

### 3.8. Metabolic Pathway Analysis

**Figure 6:**
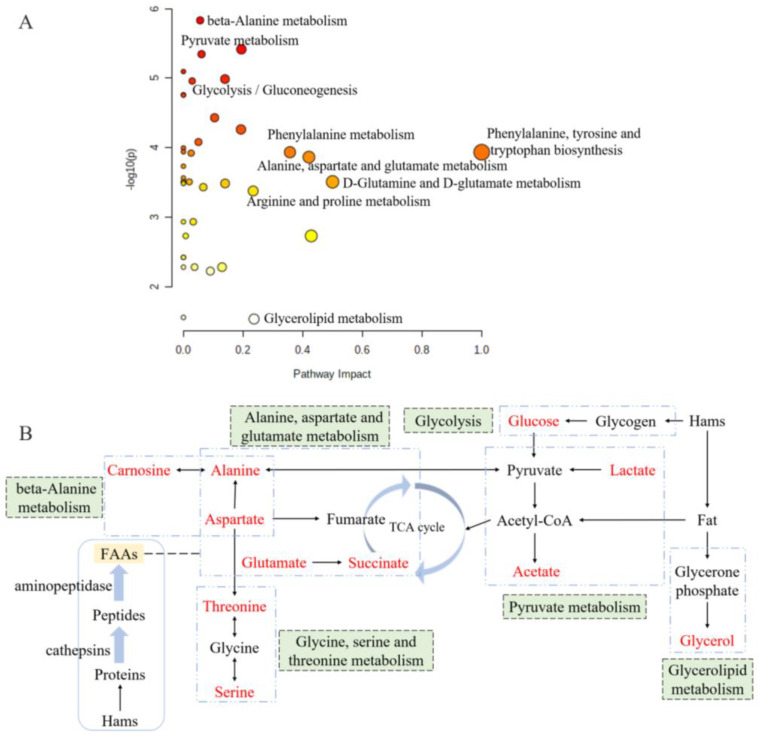
Overview of pathway analysis for significantly differential metabolites (**A**) and the metabolic pathway network related to the key taste compounds (**B**) based on KEGG. The size and color of each circle is based on metabolic pathway impact value and *p* value. Metabolites in red represent the key taste compounds in this work.

Metabolic pathway analysis is a great approach for evaluating the relationship between metabolites, and it could reconstruct the networks of biochemical reaction [39,40]. According to the metabolic pathway analysis of differently abundant metabolites between the groups of 0% and 2% potassium lactate, a total of 39 correlated metabolic pathways were found and visualized in Figure 6A, and the impact value of 18 pathways was higher than 0.05. Based on the KEGG database, the six metabolic pathways are summarized and presented in Figure 6B, and they were pyruvate metabolism, glycine, serine and threonine metabolism, alanine, aspartate and glutamate metabolism, beta-alanine metabolism, glycolysis and glycerolipid metabolism. These six pathways included all the significantly differential metabolites identified by PLS-DA and taste analysis. Thus, it could be considered that they were the main pathways significantly influenced by potassium lactate and were closely connected with the taste development of Rugao ham cured with 2% potassium lactate. The six metabolic pathways were closely related to alanine, aspartate and glutamate (Figure 6B). Aspartate and alanine were linked to beta-alanine metabolism and alanine, aspartate and glutamate metabolism; alanine was also associated with pyruvate metabolism and alanine, aspartate and glutamate metabolism; while aspartate was a connection between alanine, aspartate and glutamate metabolism and glycine, serine and threonine metabolism. Furthermore, glucose and glycerol were the products from glycogen and fat degradation. Therefore, three of these six pathways would be classified to amino acids metabolic pathways, proving that amino acids metabolism was the main pathway significantly influenced by potassium lactate.

## 4. Conclusions

This study proves that the addition of potassium lactate plays an important role in the improvement of the taste of Rugao ham. Aspartate, glutamate, alanine, serine, threonine, acetate, lactate, succinate, carnosine, β-glucose and glycerol had the most intense response in developing the taste of Rugao ham salted with 2% potassium lactate. Furthermore, the pathway of amino acids metabolism was responsible for the taste development of Rugao ham. The study provides an important basis for meat processors to potentially improve the taste quality of Chinese dry-cured ham by the treatment of potassium lactate.

## Figures and Tables

**Table 1:** Metabolite content of Rugao ham (mg/g) and taste active values (TAVs).

		Potassium Lactate Levels						*p*-Value
		0%		1%		2%		
		Content	TAVs	Content	TAVs	Content	TAVs	
Free amino acids	Phenylalanine	1.64 ± 0.18 ^b^	1.82	2.02 ± 0.49 ^ab^	2.25	2.77 ± 0.43 ^a^	3.08	<0.01
	Alanine	4.37 ± 0.75 ^c^	7.28	6.32 ± 1.30 ^b^	10.53	8.03 ± 1.46 ^a^	13.38	<0.001
	Methionine	0.17 ± 0.01 ^b^	0.56	0.16 ± 0.03 ^b^	0.54	0.22 ± 0.01 ^a^	0.74	<0.05
	Glutamate	2.89 ± 0.44 ^b^	9.62	4.22 ± 1.00 ^ab^	14.05	5.26 ± 0.99 ^a^	17.53	<0.01
	Arginine	0.07 ± 0.05 ^b^	0.13	0.10 ± 0.04 ^b^	0.21	0.18 ± 0.07 ^a^	0.36	<0.05
	Lysine	2.34 ± 0.44 ^b^	4.67	2.90 ± 0.57 ^ab^	5.79	3.52 ± 0.64 ^a^	7.05	<0.01
	Tyrosine	0.19 ± 0.01 ^b^	0.21	0.36 ± 0.08 ^a^	0.40	0.33 ± 0.06 ^a^	0.37	<0.05
	Leucine	2.42 ± 0.39 ^b^	1.27	3.05 ± 0.61 ^a^	1.61	3.39 ± 0.49 ^a^	1.78	<0.05
	Taurine	1.17 ± 0.17 ^b^	0.09	1.46 ± 0.29 ^b^	0.12	1.86 ± 0.36 ^a^	0.15	<0.05
	Proline	0.59 ± 0.10 ^b^	0.20	0.79 ± 0.24 ^ab^	0.26	1.01 ± 0.21 ^a^	0.34	<0.01
	Serine	1.00 ± 0.17 ^b^	0.67	1.54 ± 0.22 ^a^	1.02	1.87 ± 0.45 ^a^	1.24	<0.05
	Threonine	2.28 ± 0.20 ^b^	0.88	3.27 ± 0.63 ^a^	1.26	3.78 ± 0.45 ^a^	1.45	<0.05
	Aspartate	3.30 ± 0.19 ^c^	3.30	3.91 ± 0.57 ^b^	3.91	5.37 ± 0.52 ^a^	5.37	<0.001
	Valine	1.30 ± 0.15 ^b^	3.25	1.61 ± 0.27 ^b^	4.02	2.16 ± 0.39 ^a^	5.40	<0.05
	Isoleucine	2.12 ± 0.24 ^c^	2.35	2.89 ± 0.60 ^b^	3.21	3.93 ± 0.25 ^a^	4.37	<0.001
	Total	25.84 ± 3.10 ^c^		34.59 ± 6.84 ^b^		43.67 ± 6.43 ^a^		<0.05
Peptides	Anserine	0.44 ± 0.04 ^c^	0.15	0.59 ± 0.09 ^b^	0.19	0.69 ± 0.05 ^a^	0.23	<0.01
	Carnosine	4.47 ± 0.35 ^c^	0.84	5.69 ± 0.88 ^b^	1.07	7.10 ± 0.52 ^a^	1.34	<0.001
	Total	4.91 ± 0.39 ^c^		6.28 ± 0.97 ^b^		7.79 ± 0.56 ^a^		<0.001
Organic acids	Acetate	1.32 ± 0.03 ^b^	6.59	2.16 ± 0.42 ^a^	10.82	2.45 ± 0.37 ^a^	12.24	<0.05
	Butyrate	0.18 ± 0.05 ^c^	0.51	0.75 ± 0.41 ^b^	2.14	1.26 ± 0.36 ^a^	3.61	<0.001
	Succinate	0.55 ± 0.15 ^b^	5.21	0.96 ± 0.13 ^a^	9.02	0.84 ± 0.05 ^a^	7.94	<0.05
	Creatine	2.72 ± 0.23 ^b^	0.35	2.99 ± 0.39 ^b^	0.38	3.49 ± 0.45 ^a^	0.44	<0.05
	Formate	0.23 ± 0.04 ^b^	0.77	0.30 ± 0.07 ^a^	1.01	0.36 ± 0.05 ^a^	1.21	<0.05
	Lactate	8.00 ± 0.62 ^c^	6.35	12.53 ± 2.23 ^b^	9.94	14.77 ± 1.77 ^a^	11.73	<0.001
	Total	13.00 ± 0.91 ^c^		19.69 ± 3.24 ^b^		23.18 ± 2.84 ^a^		<0.001
Nucleic acids	AMP	0.11 ± 0.01	0.21	0.11 ± 0.01	0.23	0.13 ± 0.02	0.26	>0.05
	Inosine	0.18 ± 0.03 ^b^	0.07	0.24 ± 0.03 ^a^	0.09	0.26 ± 0.01 ^a^	0.10	<0.05
	Uridine	0.03 ± 0.01	NA	0.03 ± 0.01	NA	0.05 ± 0.01	NA	>0.05
	Uracil	0.11 ± 0.02 ^b^	NA	0.15 ± 0.02 ^a^	NA	0.14 ± 0.02 ^a^	NA	<0.05
	Total	0.42 ± 0.05 ^b^		0.52 ± 0.07 ^a^		0.58 ± 0.06 ^a^		<0.05
Other	β-glucose	4.61 ± 0.63 ^b^	0.28	6.50 ± 1.31 ^a^	0.40	7.11 ± 1.18 ^a^	0.44	<0.01
	myo-Inositol	0.51 ± 0.10 ^b^	NA	0.62 ± 0.11 ^ab^	NA	0.70 ± 0.09 ^a^	NA	<0.05
	Choline	1.19 ± 0.17 ^b^	2.86	1.62 ± 0.32 ^a^	3.87	1.72 ± 0.13 ^a^	4.12	<0.01
	Betaine	0.50 ± 0.06 ^b^	NA	0.53 ± 0.13 ^b^	NA	0.91 ± 0.14 ^a^	NA	<0.05
	Glycerol	5.31 ± 1.11 ^b^	NA	7.04 ± 0.98 ^a^	NA	6.58 ± 0.51 ^ab^	NA	<0.01
	Creatinine	0.48 ± 0.05 ^b^	0.42	1.07 ± 0.41 ^b^	0.94	1.96 ± 0.72 ^a^	1.73	<0.01
	Nicotinamide	0.38 ± 0.02 ^b^	NA	0.49 ± 0.09 ^ab^	NA	0.59 ± 0.15 ^a^	NA	<0.05
	Total	7.86 ± 1.35 ^b^		10.74 ± 1.91 ^a^		11.76 ± 1.55 ^a^		<0.01

Metabolite content was expressed as mean value ± standard deviation (SD) obtained from six replicates of each group. NA indicates no available threshold value of taste; ^a–c^ different lowercase letters mean significant difference among three groups.

## Data Availability

The data presented in this study are available on request from the corresponding author.

## Supplementary Materials

The following supporting information can be downloaded at: https://www.mdpi.com/article/10.3390/foods12071453/s1, Figure S1: ^1^H NMR spectra of Rugao ham samples treated with 0% potassium lactate (A), 1% potassium lactate (B) and 2% potassium lactate (C); Table S1: Optical density of protein bands in the SDS-PAGE detected in Rugao hams; Table S2: Summary of identified and analyzed metabolites in Rugao ham samples and their numbers on ^1^H NMR spectra.
