# Supplementary material for: 1H NMR Reveals the Mechanism of Potassium Lactate on Proteolysis and Taste Metabolites of Rugao Ham
Source: Foods. 2023 Mar 29;12(7):1453. doi: 10.3390/foods12071453 (PMC10093880; doi:10.3390/foods12071453)
Supplement: Supplementary file 1 [file foods-12-01453-s001.zip › foods-2248921-supplementary.pdf]

Figure S1 ^{1}H NMR spectra of Rugao ham samples treated with 0% potassium lactate (A), 1% potassium lactate (B) and 2% potassium lactate (C).

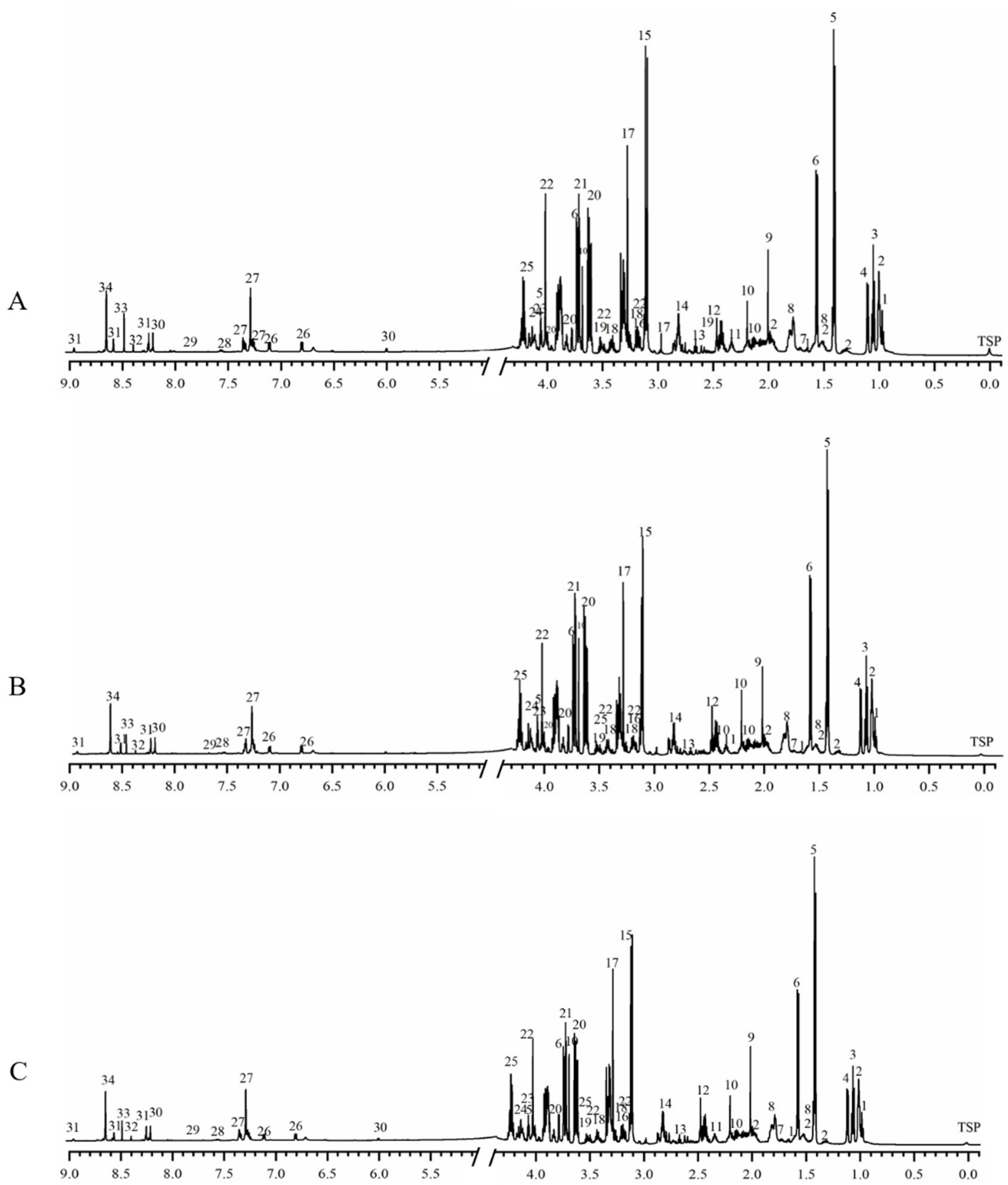

Table S1 Optical density of protein bands in the SDS-PAGE detected in Rugao hams.

	molecular weight (kDa)	Potassium lactate levels			
		0%	0.5%	1%	2%
sarcoplasmic proteins	69	203830.02±47811.98^{b}	226296.59±33608.41^{ab}	245713.69±24049.31^{ab}	292841.3±30874.3^{a}
	63	370699.08±54170.92^{b}	391973.14±70603.86^{ab}	447796.13±9602.87^{ab}	468070.06±9958.94^{a}
	56	353430.35±34954.65^{a}	321653.4±42123.78^{a}	323524.5±3635.5^{a}	320711.44±2813.44^{a}
	43	322632.12±39875.88^{a}	325176.02±22537^{a}	301738.5±44173.5^{a}	277955.35±41117.65^{a}
	37	325052.7±15478.7^{a}	236784.91±37387.09^{b}	228831.63±26259.37^{b}	204386.88±48226.12^{b}
	26	309473.47±38249.53^{a}	242445.08±24091.88^{b}	228297.84±20133.34^{b}	189743.75±27106.25^{b}
	18	275475±18805.37^{a}	255146.33±14638.76^{a}	224830.67±4632.88^{b}	218493.67±10131.51^{b}
myofibrillar proteins	100	114533.71±16252.05^{a}	129693.89±25777.95^{a}	134971.33±1019.67^{a}	147431.43±24945.05^{a}
	70	79749.45±28845.55^{a}	54637.81±24492.81^{ab}	20334.20±5228.80^{b}	28118.77±9019.23^{b}
	65	173657.69±6946.31^{a}	82793.12±12398.41^{b}	87532.48±5889.24^{b}	77396.00±7036.00^{b}
	43	250270.40±14105.43^{b}	262050.88±9215.30^{ab}	250681.79±12776.44^{b}	283424.16±10588.80^{a}
	36	83305.51±22181.57^{a}	84706.67±1041.87^{a}	81204.07±6613.50^{a}	85734.53±1242.53^{a}
	33	60736.00±11680.00^{a}	70025.69±2598.68^{a}	70803.23±4592.77^{a}	70589.88±8208.13^{a}
	26	58725.56±3595.44^{b}	72350.57±6318.43^{a}	77901.07±1277.07^{a}	77172.69±1846.40^{a}

Optical density was expressed as mean values ± standard deviation (SD) obtained from three replicates of each group. $^{a–b}$ different lowercase letters mean significant difference among groups.

ND: Not detected.

Table S2 Summary of the identified and analyzed metabolites in Rugao ham samples and their numbers on ^{1}H NMR spectra.

No.	Metabolite	$\delta^{1}H$(ppm) and multiplicity
1	Butyrate	0.88(t), 1.54(m)
2	Isoleucine	3.67(d), 1.98(m), 1.26(m), 1.48(m), 1.01(d), 0.94(t)
3	Leucine	3.74(m), 1.73(m), 1.69(m), 0.98(d), 0.96(d)
4	Valine	3.62(d), 2.28(m), 0.99(d), 1.04(d)
5	Lactate	4.13(q), 1.33(d)
6	Alanine	3.79(q), 1.49(d)
7	Arginine	1.92(m), 1.66(m), 3.26(t), 3.76(t)
8	Lysine	3.76(t), 3.03(t), 1.92(m), 1.73(m), 1.48(d)
9	Acetate	1.92(s)
10	Glutamate	3.77(m), 2.36(dt), 2.12(m)
11	Proline	4.1(m),3.4(dt), 2.1(m)
12	Succinate	2.41(s)
13	Methionine	3.85(dd), 2.15(m), 2.65(t), 2.14(s)
14	Aspartate	3.92(dd), 2.80(dd), 2.70(dd)
15	Creatine	3.04(s), 3.93(s)
16	Creatinine	4.06(s), 3.05(s)
17	Carnosine	8.50(s), 7.19(s), 3.21(m)
18	Taurine	3.44(t), 3.29(t)
19	Glycerol	3.57(dd), 3.67(dd), 3.80(dd)
20	β-glucose	3.91(dd), 3.76(m), 3.42(m)
21	myo-Inositol	4.12(dd), 3.75(t)
22	Choline	4.07(d), 3.53(d), 3.21(s)
23	Betaine	3.91(s), 3.27(s)
24	Serine	4.0(dd), 3.9(dd)
25	Threonine	4.26(m), 3.58(d)
26	Tyrosine	7.2(d), 6.90(d)
27	Phenylalanine	7.33(q), 7.43(t), 7.38(m)
28	Uracil	7.54(d), 5.81(d)
29	Uridine	5.9(m), 7.9(m)
30	Inosine	8.23(s), 8.34(s), 6.10(d)
31	Nicotinamide	8.95(d), 8.62(dd), 8.26(m)
32	Formate	8.46(s)
33	AMP	8.23(s), 8.59(s), 6.15(d)
34	Anserine	8.63(s), 7.28(s), 4.51(dd), 3.77(s)
